# Supplementary material for: Immunity and Protective Efficacy of a Plant-Based Tobacco Mosaic Virus-like Nanoparticle Vaccine against Influenza a Virus in Mice
Source: Vaccines (Basel). 2024 Sep 26;12(10):1100. doi: 10.3390/vaccines12101100 (PMC11510914; doi:10.3390/vaccines12101100)
Supplement: Supplementary file 1 [file vaccines-12-01100-s001.zip › vaccines-3154986-supplementary.pdf]

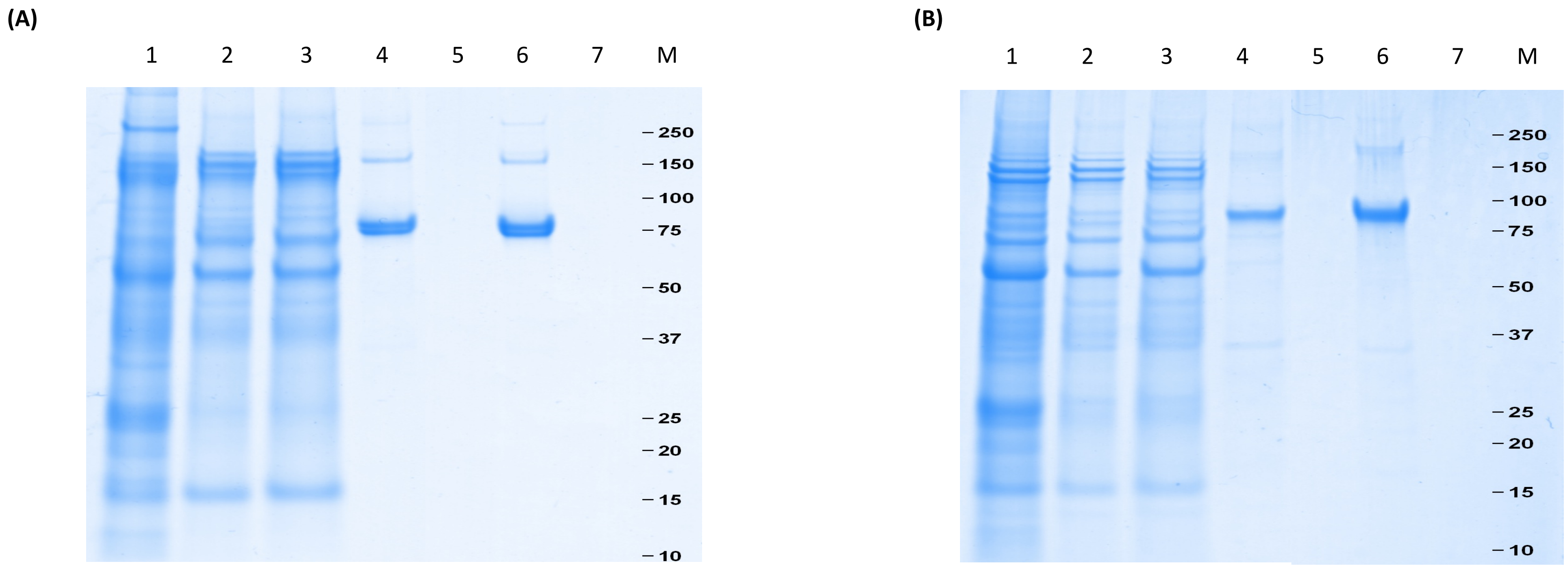

Supplementary Figure S1. The HA-con protein purification profiles. The HA-con proteins were purified from plants transfected with H1-con (pKB111) and H3-con (pKBP117). Reducing SDS-PAGE analysis was used to assess the purity of (**A**) H1-con and (**B**) H3-con, respectively. Lanes 1: Crude plant extracts; Lanes 2: Clarified extract; Lanes 3: Prepared extract for loading on TALON metal affinity column; Lanes 4: TALON eluant; Lanes 5: Blank; Lanes 6: Final products after Capto-Q anion exchange and diafiltration; Lanes 7: Wash flowthrough; M: Protein marker.

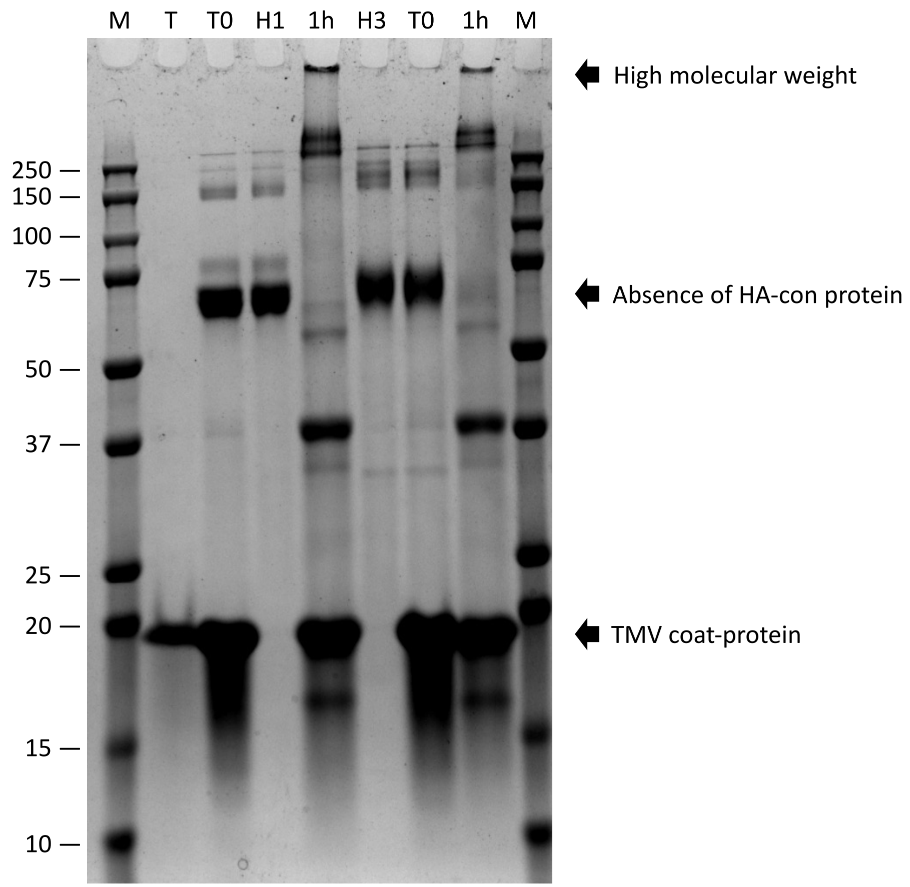

Supplementary Figure S2. Conjugation of HA-con proteins to TMV. Samples were overloaded to visualize the presence of HA-con protein in the conjugation which was used as the vaccine. 5 µg of HA-con (coat protein) or 10 µg of TMV-HA mixture (conjugate) was separated on a BioRad TGX Criterion gel (8-16%) and stained with Coomassie Brilliant blue (R-250). M: Protein marker (BioRad Dual color); T: TMV coat protein, T0: Mixture of TMV and HA-con protein prior to addition of EDC; H1- or H3-con proteins, 1h: TMV-HA conjugates after 1 hour of reaction in 4 mM EDC/NHS at pH 5. Conjugation reactions were considered complete as determined by the absence of free HA-con protein.

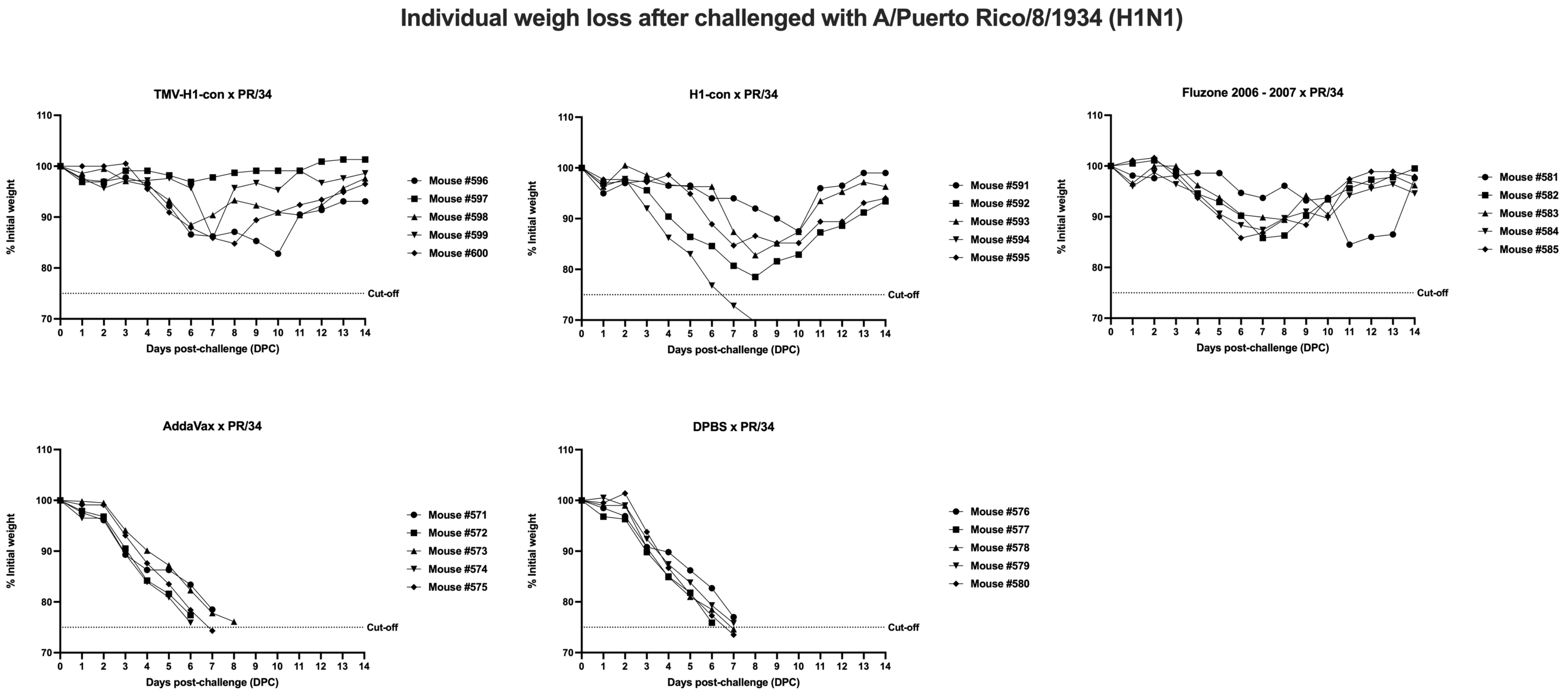

Supplementary Figure S3. Individual wight loss after challenged with A/Puerto Rico/8/1934 (H1N1) virus. Mice were monitored for weight loss over 14 days and animals that showed 25% weight loss were humanely euthanized.

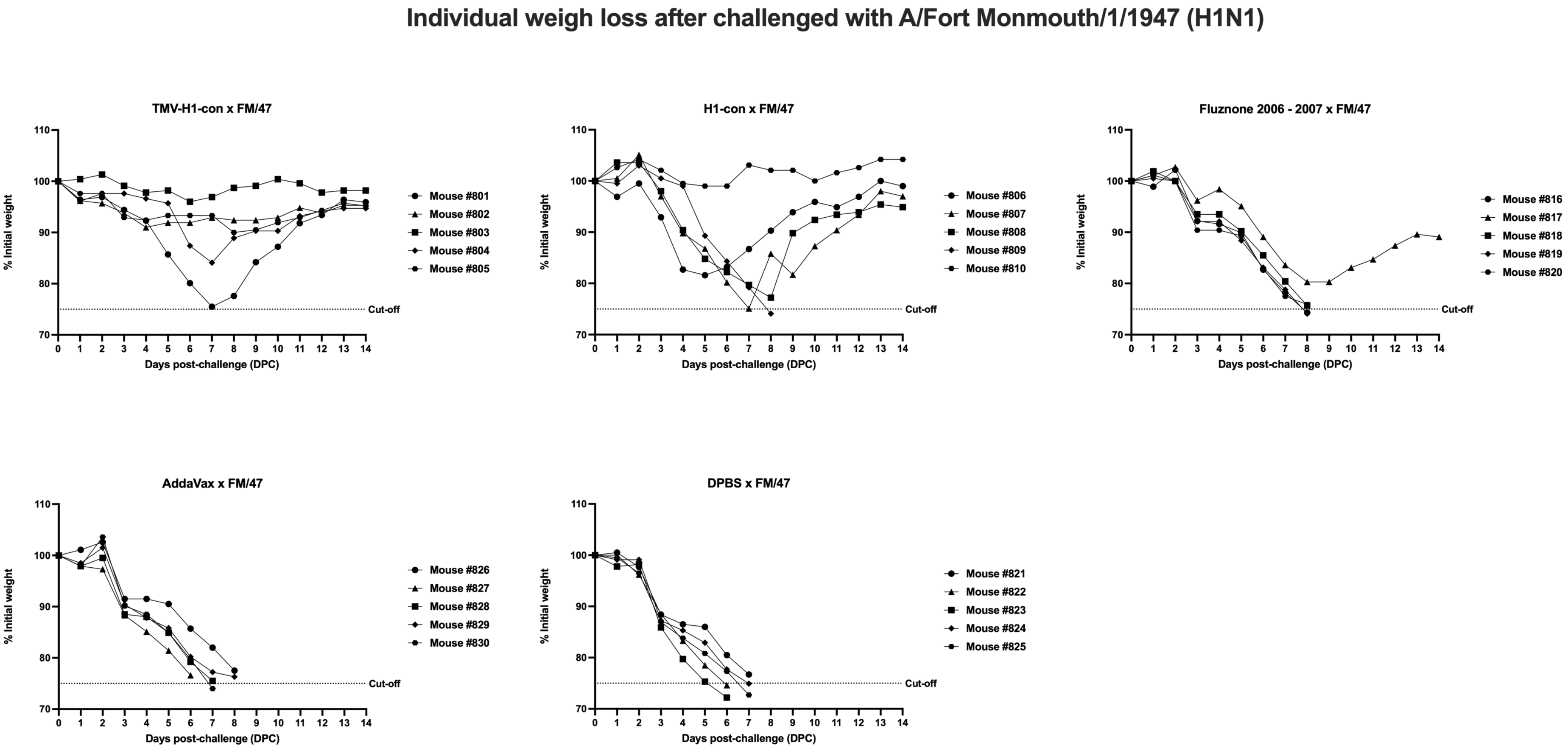

Supplementary Figure S4. Individual wight loss after challenged with A/Fort Monmouth/1/1947 (H1N1) virus. Mice were monitored for weight loss over 14 days and animals that showed 25% weight loss were humanely euthanized.

Individual weigh loss after challenged with A/Texas/1/1977 (H3N1)

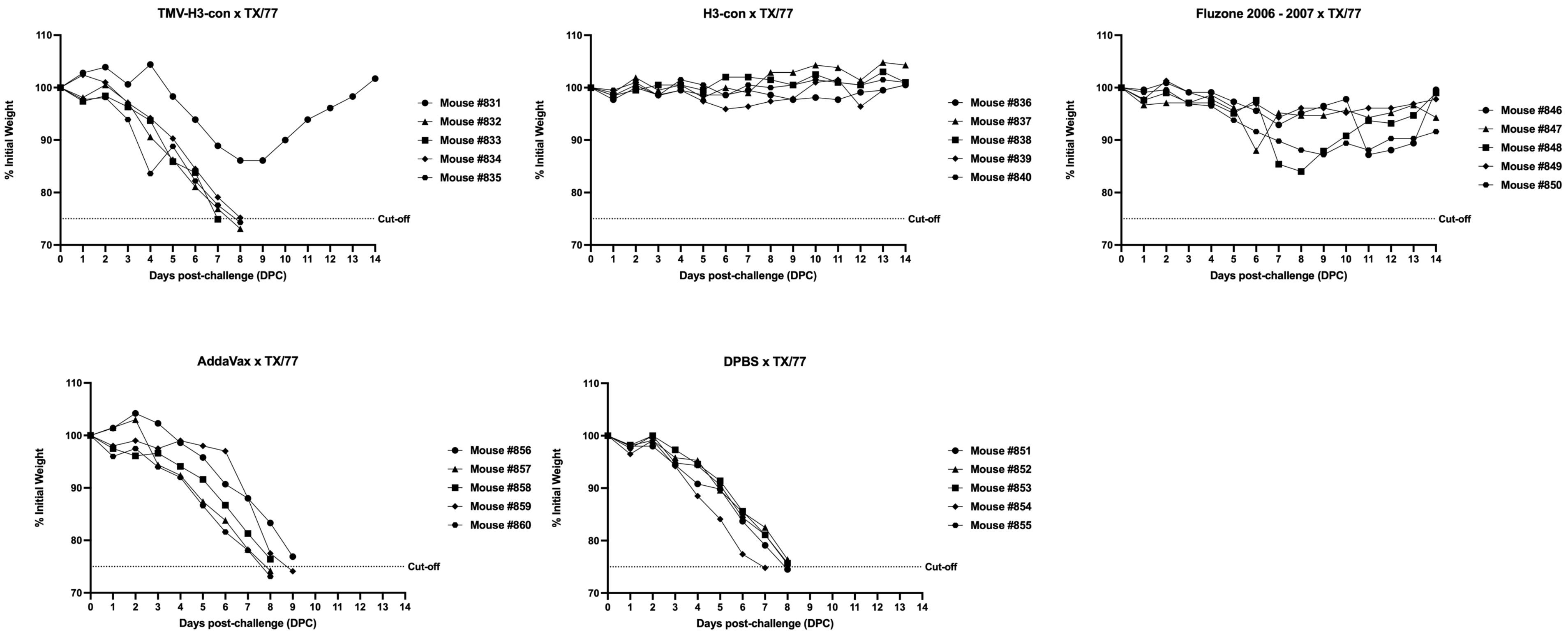

Supplementary Figure S5. Individual wight loss after challenged with A/Texas/1/1977 (H3N1) virus. Mice were monitored for weight loss over 14 days and animals that showed 25% weight loss were humanely euthanized.

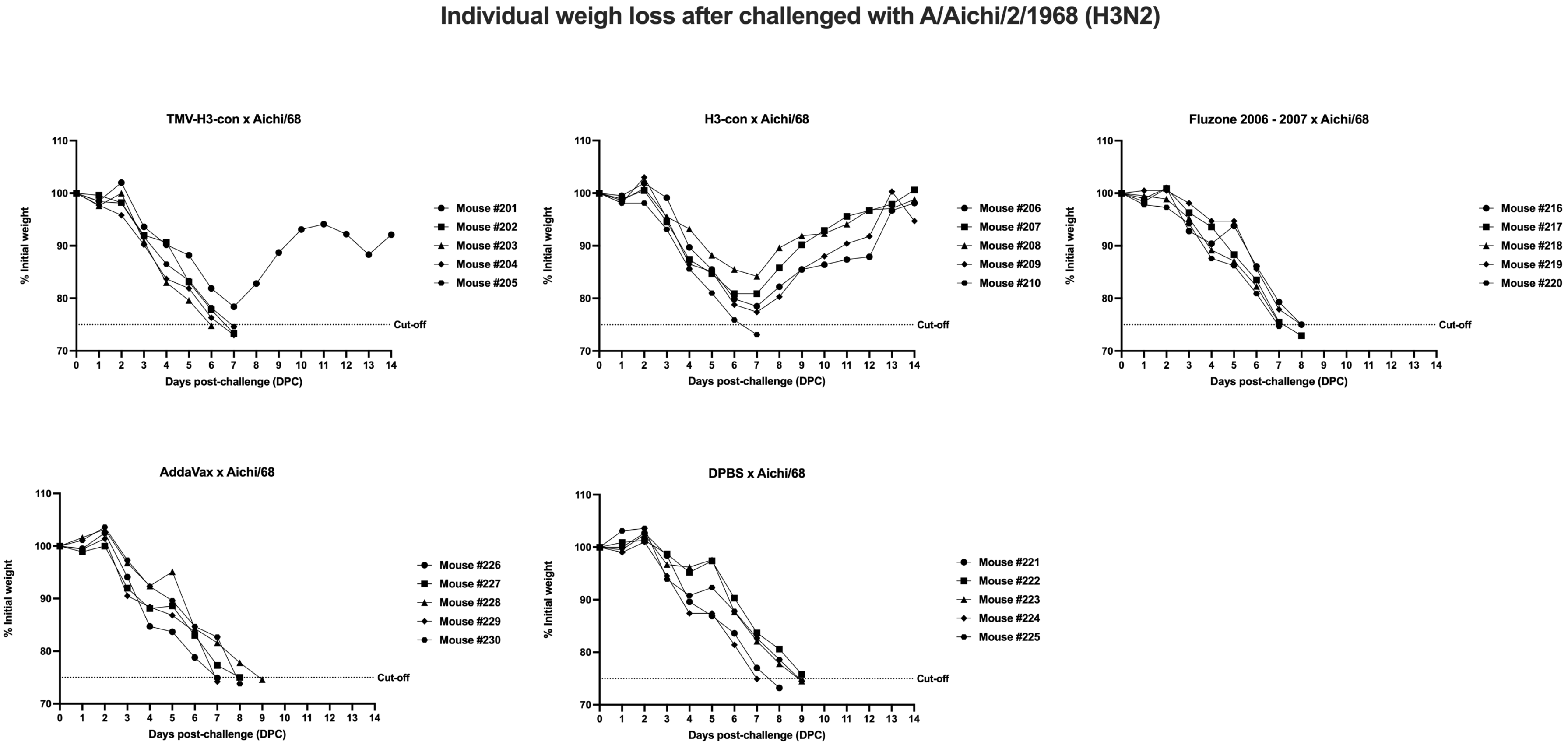

Supplementary Figure S6. Individual wight loss after challenged with A/Aichi/2/1968 (H3N2) virus. Mice were monitored for weight loss over 14 days and animals that showed 25% weight loss were humanely euthanized.
